# SGLT2 inhibitors and exercise in diabetic kidney disease: mechanistic rationale, emerging clinical evidence, and future perspectives

**DOI:** 10.3389/fmed.2026.1968588

**Published:** 2026-10-08

**Authors:** Huajuan Li, Min Ai, Xiangyu He, Caijun Liu, Xuegui Ju

**Affiliations:** 1 Department of Clinical Nutrition, Gaoxian People's Hospital, Yibin, China; 2 Department of Neurology, Gaoxian People's Hospital, Yibin, China; 3 Department of Cardiac and Thoracic Aortic Surgery, Christian Doppler Laboratory for Mechanical Circulatory Support, Medical University of Vienna, Vienna, Austria; 4 Department of Cardiac Surgery, Southwest Hospital of AMU, Chongqing, China; 5 Department of Intensive Care Unit, Gaoxian People's Hospital, Yibin, China; 6 Department of General Practice, The First Affiliated Hospital of Chengdu Medical College, Chengdu, China; 7 Department of General Practice, Clinical Medical College, Chengdu Medical College, Chengdu, China; 8 Department of General Practice, Chengdu University of Traditional Chinese Medicine, Chengdu, China

**Keywords:** diabetic kidney disease, exercise therapy, inflammatory response, renal protection, SGLT2 inhibitors

## Abstract

Diabetic kidney disease (DKD) is a major complication of diabetes and a leading cause of chronic kidney disease and kidney failure worldwide. Despite substantial advances in pharmacological therapy, residual risks of renal and cardiovascular disease remain considerable, underscoring the need for complementary therapeutic strategies. Sodium-glucose cotransporter 2 (SGLT2) inhibitors have emerged as cornerstone therapies for DKD because of their glucose-independent renoprotective and cardioprotective effects. Exercise, meanwhile, provides broad metabolic, cardiovascular, anti-inflammatory, and antioxidant benefits and is recommended as an integral component of diabetes and chronic kidney disease management. Increasing evidence suggests that these two interventions may exert complementary effects on several interconnected pathways involved in DKD progression, including glomerular hyperfiltration, metabolic dysregulation, oxidative stress, inflammation, mitochondrial dysfunction, and renal fibrosis. This review summarizes the pathophysiological mechanisms underlying DKD and examines the mechanistic rationale for integrating SGLT2 inhibitors with exercise. We further discuss emerging experimental and clinical evidence regarding their potential effects on renal function, metabolic control, cardiovascular health, and exercise capacity, together with important safety considerations and challenges in individualized exercise prescription. Although available evidence provides a biologically plausible basis for potentially additive interactions between these interventions, direct clinical evidence supporting the superiority of combined therapy over either intervention alone remains limited. We therefore highlight key knowledge gaps, including the lack of adequately powered randomized controlled trials, standardized exercise protocols, and validated biomarkers for monitoring treatment response. Future studies should establish the efficacy, safety, and optimal implementation of combined SGLT2 inhibitor and exercise strategies across different stages and phenotypes of DKD, thereby facilitating their translation into individualized cardiorenal care.

## 1 Introduction

Diabetic kidney disease (DKD), previously referred to as diabetic nephropathy (DN), is one of the most prevalent and devastating microvascular complications of diabetes mellitus and remains the leading cause of chronic kidney disease (CKD) and end-stage kidney disease (ESKD) worldwide (1). As the global prevalence of diabetes continues to rise, the burden of DKD has increased substantially, resulting in considerable morbidity, mortality, and healthcare expenditures (2). Despite advances in glycemic control and renin–angiotensin system (RAS) blockade, a substantial proportion of patients continue to experience progressive renal function decline (3), highlighting the need for more effective therapeutic strategies.

The introduction of sodium-glucose cotransporter-2 (SGLT2) inhibitors has fundamentally transformed the management of DKD. Initially developed as glucose-lowering agents, SGLT2 inhibitors have consistently demonstrated robust renoprotective and cardioprotective effects that extend beyond glycemic control. Large-scale randomized clinical trials, including DAPA-CKD, CREDENCE, and EMPA-KIDNEY, have shown that SGLT2 inhibitors significantly reduce albuminuria, slow the decline in estimated glomerular filtration rate (eGFR), delay progression to kidney failure, and decrease cardiovascular events in patients with or without diabetes (4). These findings have led to a paradigm shift in DKD management, with current international guidelines recommending SGLT2 inhibitors as a cornerstone therapy for patients with diabetes and chronic kidney disease (5).

Alongside pharmacological therapy, lifestyle modification remains an essential component of comprehensive DKD management. Regular exercise has been shown to improve glycemic control, enhance insulin sensitivity, reduce blood pressure, attenuate chronic inflammation and oxidative stress, improve endothelial function, and enhance cardiorespiratory fitness. These beneficial effects target several of the key pathophysiological mechanisms involved in DKD progression. Consequently, major clinical guidelines consistently recommend structured physical activity as part of routine diabetes management. Nevertheless, exercise interventions remain markedly underutilized in clinical practice. Barriers including poor patient adherence, limited access to supervised exercise programs, concerns regarding hypoglycemia, cardiovascular complications, reduced physical capacity, and the absence of individualized exercise prescriptions contribute to the gap between guideline recommendations and real-world implementation.

Emerging evidence suggests that combining SGLT2 inhibitors with exercise may provide complementary or potentially additive renoprotective benefits. Although both interventions improve metabolic control and cardiovascular health, they influence disease progression through partially distinct yet complementary mechanisms. SGLT2 inhibitors primarily restore tubuloglomerular feedback, reduce intraglomerular pressure, promote natriuresis, and improve renal energy metabolism, whereas exercise enhances insulin sensitivity, improves mitochondrial function, suppresses chronic inflammation, and increases antioxidant capacity. Their complementary actions on renal hemodynamics, metabolic regulation, oxidative stress, inflammation (6), endothelial dysfunction, and tissue remodeling suggest the potential for enhanced therapeutic efficacy beyond either intervention alone. Furthermore, improvements in exercise tolerance and cardiovascular function associated with SGLT2 inhibitor therapy may facilitate greater participation in physical activity, thereby creating a positive therapeutic feedback loop.

Despite these promising mechanistic interactions, the current evidence remains fragmented. Most published reviews have examined the renoprotective mechanisms and clinical benefits of SGLT2 inhibitors or exercise therapy independently. Reviews focusing specifically on their combined application in DKD are scarce, and the potential biological interactions, clinical evidence, safety considerations, and remaining knowledge gaps have not been comprehensively synthesized. Moreover, while numerous experimental studies have explored overlapping molecular pathways, including oxidative stress, inflammation, mitochondrial dysfunction, fibrosis, and autophagy, the extent to which these pathways contribute to the hypothesized additive effects of combination therapy remains insufficiently understood.

Throughout this review, we use “additive” to describe a combined effect equal to the sum of the two independent effects, and “synergistic” to describe a combined effect greater than the sum of the independent effects—a strictly stronger claim requiring formal demonstration of a statistical or biological interaction (7). Because no study to date has formally tested an SGLT2 inhibitor×exercise interaction in DKD, claims of synergy are not made anywhere in this review; where the mechanisms of the two interventions converge on the same pathway, the terms “complementary” or “potentially additive” are used instead.

## 2 Literature search methodology

This is a narrative review. We searched PubMed/MEDLINE, Web of Science, Scopus, and the Cochrane Library for articles published from January 2015 to August 2026, using combinations of the search terms: “SGLT2 inhibitors” (and individual agent names: dapagliflozin, empagliflozin, canagliflozin, ipragliflozin, tofogliflozin), “exercise/exercise training/physical activity”, and “diabetic kidney disease/diabetic nephropathy/chronic kidney disease”. Reference lists of included articles and relevant reviews were manually screened for additional publications. English-language original research articles (randomized controlled trials, observational studies, and animal/*in-vitro* experiments) and consensus or guideline documents relevant to SGLT2 inhibitor therapy, exercise intervention, or their combination in DKD were eligible. Because direct combination evidence is scarce, no study design was excluded *a priori*; instead, each study's design and population are explicitly identified at first mention, and human clinical evidence is consistently distinguished from preclinical evidence throughout the text. The search and selection were performed independently by two authors (H.L. and M.A.), with discrepancies resolved by the corresponding author (X.J.).

## 3 Pathophysiology of diabetic kidney disease

### 3.1 Glomerular hemodynamic dysfunction

Studies have shown that under hyperglycemic conditions, estimated glomerular filtration rate (eGFR) is significantly elevated. The state of glomerular hyperfiltration increases the burden on the kidney, primarily manifesting as tubular overload, proteinuria, and tubular injury, thereby promoting the progression of diabetic kidney disease (8)^.^ Concurrently, in patients with diabetes, activation of the renin-angiotensin-aldosterone system (RAAS) increases glomerular pressure and filtration rate, similarly leading to renal fibrosis and inflammation. RAAS activation further impairs tubular function through pro-inflammatory pathways, forming a vicious cycle that exacerbates glomerular injury and drives DKD progression (9).

### 3.2 Metabolic dysregulation

Hyperglycemia is a common pathological state in patients with diabetes, which can directly cause kidney damage and exacerbate renal injury through multiple mechanisms. Retrospective follow-up studies have shown that hyperglycemia leads to glomerular basement membrane thickening, mesangial cell proliferation, and glomerulosclerosis, thereby impairing glomerular filtration function and resulting in chronic kidney injury and functional decline (10). Simultaneously, prolonged hyperglycemia also induces tubular cell dysfunction and increases tubular stress responses, further aggravating kidney damage (11, 12). Secondly, the formation of advanced glycation end products (AGEs) is closely associated with hyperglycemia. AGEs can directly damage tubules and glomeruli, and can also promote local inflammatory responses by binding to transmembrane AGE receptors (RAGE) (13),activating oxidative stress and inflammatory reactions, stimulating macrophages and other immune cells (14), and inducing the release of renal inflammatory cytokines such as tumor necrosis factor-alpha (TNF-α) and interleukin-6 (IL-6), thereby increasing the renal inflammatory burden (11, 12) and further promoting kidney injury.

### 3.3 Oxidative stress and mitochondrial dysfunction

Furthermore, under hyperglycemic conditions, reactive oxygen species (ROS) production is increased, leading to intracellular redox imbalance. Oxidative stress not only damages tubular and glomerular cells but also activates inflammatory pathways, exacerbating apoptosis and necrosis, ultimately resulting in declining renal function (13, 14). In a diabetic rat model, hyperglycemia was observed to induce tubular cell injury and dysfunction by activating the Sirt1/NF-*κ*B/miR-29/Keap1 oxidative stress-related pathway (15). The interaction of these mechanisms collectively promotes the initiation and progression of DKD.

### 3.4 Inflammatory response and immune injury

During DKD progression, inflammatory cytokines such as TNF-α and IL-6 can disrupt the glomerular filtration barrier and lead to glomerular fibrosis. Elevated IL-6 levels can directly cause deterioration of renal function (16, 17). Studies have demonstrated that the chronic inflammatory state in patients with diabetes results in abnormal immune system activation. TNF-α promotes renal inflammatory responses by activating the nuclear factor-kappa B (NF-*κ*B) pathway, exacerbating tubular injury and fibrosis, thereby accelerating the renal damage process (18, 19). Moreover, chronic low-grade inflammation can also lead to systemic immune dysregulation. Studies have revealed that the low-grade inflammation in DKD patients manifests as a polarization state characterized by increased M1 macrophages and decreased M2 macrophages, a process that intensifies renal immune injury (20, 21).

### 3.5 Renal fibrosis and progressive nephron loss

Thus, the pathogenesis of DKD results from the three-dimensional interaction of hemodynamic, metabolic, and inflammatory disturbances. First, hyperglycemia activates RAAS, leading to glomerular hypertension, hyperfiltration, and vascular endothelial injury. Sustained glomerular hypertension directly damages podocytes and the basement membrane, accelerating glomerulosclerosis (22). Second, the direct toxicity of hyperglycemia promotes AGEs accumulation, activates inflammatory pathways (such as NF-*κ*B) and oxidative stress (23), activates PKC and the polyol pathway, increases ROS production, and intensifies glomerular and tubulointerstitial inflammatory responses while damaging renal cells. Inflammatory cells infiltrate renal tissue, releasing pro-inflammatory cytokines such as TNF-α and IL-6, and the cytokine network is activated, further amplifying the inflammatory cascade (24). In the above processes, RAAS activation and AGEs jointly induce oxidative stress and fibrosis. The vicious cycle formed by multiple factors accelerates renal fibrosis, leading to the development of diabetic kidney disease. As shown in Figure 1, hyperglycemia mediates oxidative stress and inflammatory responses through advanced glycation end products (AGEs), the polyol pathway, and the protein kinase C (PKC) pathway, forming a vicious cycle that accelerates renal fibrosis and drives the progression of diabetic kidney disease (DKD).

**Figure 1:**
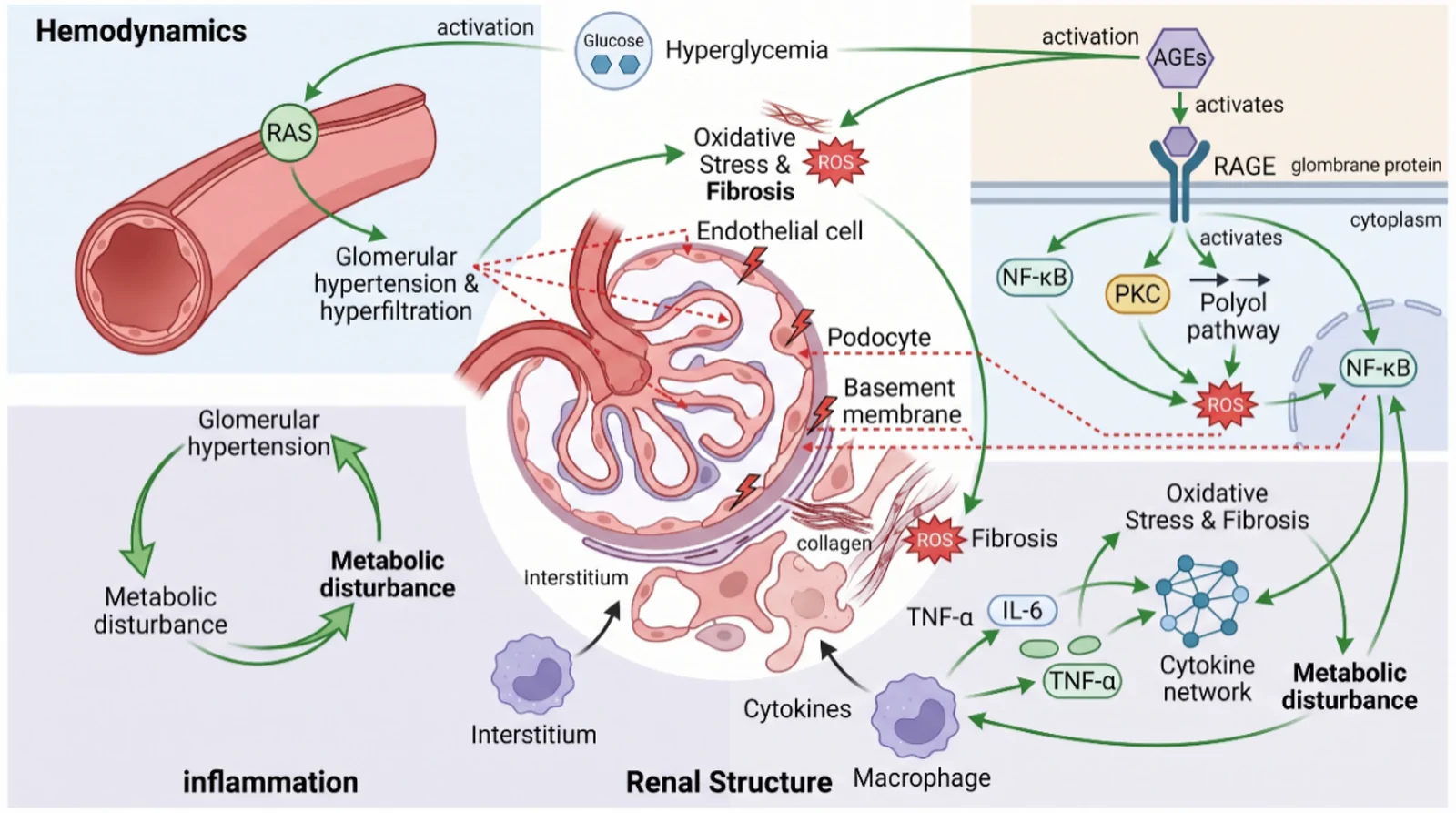
Pathophysiological mechanisms of hyperglycemia-induced renal injury. Hyperglycemia activates the RAS, AGEs, PKC, and oxidative stress pathways, resulting in glomerular hypertension, endothelial and podocyte injury, and disruption of the glomerular filtration barrier. Subsequent inflammatory activation, characterized by monocyte/macrophage infiltration and increased TNF-α and IL-6, promotes glomerulosclerosis and tubulointerstitial fibrosis. These interconnected pathways establish a vicious cycle of oxidative stress, inflammation, and renal injury.

## 4 Potential complementary effects of SGLT2 inhibitors combined with exercise therapy

### 4.1 Restoration of renal hemodynamics

Multiple experimental studies have demonstrated that SGLT2 inhibitors promote urinary sodium excretion and modulate intratubular sodium concentration, thereby influencing glomerular hemodynamics, reducing glomerular hyperfiltration, and consequently alleviating glomerular pressure and injury. Relevant studies have shown that SGLT2 inhibitors exhibit favorable renoprotective effects in both diabetic and non-diabetic patients (25, 26). Furthermore, SGLT2 inhibitors effectively reduce cardiac output and blood pressure in diabetic patients, changes that are complementary to improvements in glomerular filtration function. By lowering blood pressure, renal blood perfusion is further optimized and glomerular load is reduced, which holds significant clinical implications for DKD progression (27, 28). The American Diabetes Association guidelines (29) recommend the use of SGLT2 inhibitors or GLP-1 receptor agonists (GLP-1RA) in patients with T2DM and CKD to reduce the risk of kidney disease progression and cardiovascular disease (Level of Evidence: Grade A, assigned according to the American Diabetes Association Standards of Care in Diabetes—2024 evidence-grading system (29). Dong et al. demonstrated in a rat model of hypertensive nephropathy (30) that long-term regular exercise can lower blood pressure, improve the structure and function of hypertensive kidneys, and ameliorate RAS components, possibly by reducing oxidative stress and NF-*κ*B activity in hypertensive rat models.

### 4.2 Improvement of glucose metabolism and insulin sensitivity

SGLT2 inhibitors can significantly improve patients' glycemic control and reduce insulin resistance. Studies have shown that SGLT2 inhibitors can effectively lower blood glucose levels by promoting renal excretion of excess glucose, while also reducing body weight (31) and cardiovascular disease risk through improved metabolic status (29, 32) (Level of Evidence: Grade A). Exercise itself has also been confirmed to enhance insulin sensitivity and improve glucose metabolism (29) (Level of Evidence: Grade A). Shabab et al. (33) demonstrated in rat experiments that endurance exercise prevented diabetic cardiomyopathy through the inhibition of fibrosis and hypertrophy. Li et al. (34) reported that periodic resistance training modulated blood glucose levels in obese patients with T2DM nephropathy. In diabetes management, glycemic control is of paramount importance. Studies indicate that patients receiving SGLT2 inhibitor therapy who engage in aerobic exercise exhibit significantly lower blood glucose levels (35). Furthermore, the combination of SGLT2 inhibitors and exercise can significantly reduce insulin resistance and body weight (32, 36). Therefore, the combined intervention of SGLT2 inhibitors and exercise may produce complementary effects, enabling improved glycemic control.

### 4.3 Attenuation of inflammation

Recent studies have shown that SGLT2 inhibitors (such as dapagliflozin and empagliflozin) exert anti-inflammatory and antioxidant effects through multiple mechanisms. These agents can activate the AMPK signaling pathway and inhibit the release of pro-inflammatory cytokines, thereby attenuating inflammatory responses in renal tissue (18, 37). Research has demonstrated that SGLT2 inhibitors can counteract inflammation by inhibiting M1 macrophage activation and promoting M2 macrophage polarization, thus contributing to the restoration of immune homeostasis (18, 38), thereby also contributing to the amelioration of diabetes-related metabolic abnormalities (32).

### 4.4 Reduction of oxidative stress and mitochondrial protection

SGLT2 inhibitors can also reduce reactive oxygen species levels in renal tissue by modulating the activity of endogenous antioxidant enzymes, thereby attenuating oxidative stress-induced cellular damage (18, 31). Studies have found that exercise can lower the levels of inflammatory markers (such as TNF-α and IL-6) while increasing antioxidant enzyme activity, thus enhancing the body's capacity to resist oxidative stress (39, 40). Lass et al. (41) demonstrated in a rodent model of DKD that SGLT2i reduced oxidative stress markers in renal tissue, whereas combined therapy modulated inflammatory cytokines in skeletal muscle. Nataraj et al. (39) showed in a single-arm exercise rehabilitation study that exercise-based training improved functional capacity and renal function in patients with T2DM nephropathy. Zhou et al. (40) confirmed in db/db mice that aerobic exercise training alleviated renal injury by inhibiting Nox4-mediated NLRP3 inflammasome activation. During combined therapy, SGLT2 inhibitors exhibit positive effects in reducing tubular cell apoptosis and inhibiting inflammatory pathways, while exercise further enhances systemic anti-inflammatory capacity (42).

### 4.5 Anti-fibrotic effects and renal structural preservation

SGLT2 inhibitors directly suppress TGF-*β*1 expression and Smad2/3 phosphorylation, reducing CTGF and collagen deposition (43). In a mouse model of type 2 diabetes, dapagliflozin alleviated renal fibrosis by regulating STAT3 signaling (44). Asgari et al. (45) showed in an animal experiment that moderate-intensity interval training combined with Crataegus persica improved renal function and insulin sensitivity through the miR-126/Nrf-2 antioxidant pathway, forming a dual anti-fibrotic barrier of ‘direct blockade + indirect suppression.’

### 4.6 Improvement of cardiovascular function and exercise capacity

SGLT2 inhibitors have been shown to reduce cardiac filling pressure, improve diastolic function, and thereby enhance exercise capacity (46, 47). Multiple clinical trials have demonstrated that SGLT2 inhibitors such as dapagliflozin improve exercise capacity and quality of life in patients with heart failure (48). Moreover, for patients with DKD, exercise can improve not only physical fitness but also cardiovascular health. Studies have shown that patients engaging in aerobic and resistance training exhibit increased exercise tolerance and significantly improved cardiorespiratory endurance (49). Exercise can reduce the risk of cardiovascular events by enhancing skeletal muscle metabolic capacity and improving cardiac ejection function (50). Therefore, the combined application of SGLT2 inhibitors and exercise may produce complementary effects, further enhancing patients' overall health status. Additionally, regular exercise among patients with diabetes can significantly improve psychological well-being, reducing levels of anxiety and depression. The major cardiovascular and systemic benefits of SGLT2 inhibitors and exercise relevant to patients with DKD are summarized in Table 1. In addition, Michou et al. (49) reported that cardiopulmonary exercise testing (CPET)-guided rehabilitation exercise during maintenance hemodialysis in patients with diabetic nephropathy was associated with significant clinical benefits, effectively promoting functional recovery and improving quality of life.

**Table 1:** Complementary effects of SGLT2 inhibitors and exercise on cardiovascular function and overall health in patients with diabetic kidney disease.

Mode of Action	Optimizing Renal Hemodynamics and Function	Improving Blood Glucose Metabolic Indices	Reducing Inflammation and Oxidative Stress	Cardiovascular Benefits
SGLT2 Inhibitors	Promote sodium excretion, reduce cardiac output, lower blood pressure, improve glomerular filtration, alleviate kidney damage	Lower blood glucose, improve insulin resistance, reduce body weight	Activate AMPK pathway, inhibit inflammatory responses, improve immune balance, alleviate kidney damage	Reduce cardiac filling, improve cardiac diastolic function, reduce cardiovascular disease risk
Exercise	Long-term exercise lowers blood pressure, improves RAS components, reduces oxidative stress	Lower blood glucose, reduce body weight, alleviate insulin resistance, increase insulin sensitivity	Lower inflammatory markers, inhibit inflammasome activation, reduce oxidative stress levels, regulate immune cell activity	Enhance skeletal muscle metabolic capacity, improve cardiac pumping function, reduce cardiovascular disease risk
Common Effects	Lower blood pressure, improve hemodynamics, reduce kidney burden	Lower blood glucose, reduce body weight, alleviate insulin resistance	Reduce inflammation and oxidative stress, regulate immunity	Reduce cardiovascular disease risk

## 5 Current clinical evidence and literature review

The key clinical and preclinical evidence on SGLT2 inhibitors and/or exercise in DKD is summarized in Table 2.

**Table 2:** Summary of key clinical and preclinical evidence on SGLT2 inhibitors and/or exercise in diabetic kidney disease.

Evidence class	Study	Intervention	Population/Model	Principal findings
Human RCT	DAPA-CKD; CREDENCE; EMPA-KIDNEY	SGLT2 inhibitor (dapagliflozin, canagliflozin, empagliflozin)	CKD with and without diabetes (large DKD subgroups)	Reduced albuminuria, slowed eGFR decline, fewer renal and cardiovascular events
Human RCT (crossover)	Murillo et al. 2022	HIIT vs. moderate continuous training	Type 1 diabetes	HIIT reduced hypoglycemic events vs. continuous training
Human, open-label	Ito et al. 2017	Ipragliflozin added to background diet/exercise	T2DM with early nephropathy	HbA1c decreased, weight loss, UACR decreased; hyperfiltration subgroup benefited most
Human, single-arm	Nataraj et al. 2024	Exercise-based rehabilitation	T2DM nephropathy	Improved functional capacity and renal function
Human	Li et al. 2024	Periodic resistance training	Obese T2DM nephropathy	Improved glycemic control
Animal (mouse)	Aroor et al. 2018	Empagliflozin	Mouse model of type 2 diabetes	Reduced aortic stiffness and renal injury; restored RECK expression
Animal (db/db mice)	Zhou et al. 2022	Aerobic exercise	DKD model	Inhibited Nox4-mediated NLRP3 inflammasome activation, alleviated renal injury
Animal (rat)	Lass et al. 2026	SGLT2 inhibitor + physical training (combined)	DKD model	Attenuated metabolic dysfunction and pyroptosis-mediated renal cell death
Animal (mouse)	Wang et al. 2026	Dapagliflozin	Mouse model of type 2 diabetes	Reduced renal fibrosis via STAT3 regulation
Animal (mouse/rat)	Kim et al. 2025; Liu et al. 2026	Irisin/TIF1*γ* pathway; training + vitamin D	DN models	Attenuated renal fibrosis via muscle–kidney crosstalk
Combination evidence	Lass et al. 2026 (animal); Ito et al. 2017 (human, background exercise)	SGLT2 inhibitor + exercise	DKD rat model; T2DM nephropathy patients	Only direct combination data available: preclinical benefit and indirect clinical signals; no RCT of combination vs. monotherapy

### 5.1 Evidence for SGLT2 inhibitors in DKD

In a preclinical study, single-cell sequencing analyses indicated that Hmgcs2 and Btg2 are potential therapeutic targets of sodium-glucose cotransporter 2 inhibitors for alleviating renal fibrosis in DKD (51). In a mouse experimental model by Kim et al. (52), transcriptional intermediary factor 1*γ* (TIF1*γ*) served as a multi-target therapeutic approach for DN by attenuating renal and muscular complications through direct inhibition of fibrosis and indirect myokine-mediated inter-organ crosstalk.

### 5.2 Evidence for exercise intervention

In animal models by Liu et al. (53), exercise-induced TIF1*γ*/irisin pathway activation facilitated skeletal muscle-kidney crosstalk to attenuate fibrosis, while resistance training combined with vitamin D conferred renal protection through antioxidant and anti-inflammatory mechanisms. Aroor et al. (54) found in a mouse model of type 2 diabetes that 5-week empagliflozin treatment significantly ameliorated vascular and renal injury, including reduced aortic stiffness and renal resistivity index, decreased renal tissue fibrosis, restored expression of the anti-fibrotic mediator RECK (reversion inducing cysteine rich protein with Kazal motifs), and reversed high glucose-induced RECK suppression in human proximal tubular cells.

### 5.3 Existing studies evaluating combination therapy

Ito et al. (55) demonstrated that among patients receiving diet and exercise therapy or other glucose-lowering agents (non-SGLT2i), the addition of ipragliflozin resulted in a significant reduction in HbA1c (1.0 ± 1.2%), body weight loss (2.7 ± 2.5 kg), and a decrease in median UACR from 15.5 to 12.9 mg/gCr. Subgroup analysis revealed that patients with baseline eGFR≥90 mL/min/1.73 m^2^ exhibited more pronounced improvements in UACR and eGFR, suggesting that ipragliflozin may exert renoprotective effects through amelioration of glomerular hyperfiltration. Observational data suggest that SGLT2 inhibitor use in patients with diabetes and heart failure is associated with improved clinical outcomes (56); however, direct evidence in patients with DKD undertaking structured exercise remains limited. Two animal studies (41, 57) showed that SGLT2i primarily conferred renoprotection and glucose-lowering effects, while exercise training improved skeletal muscle metabolism and inflammatory status; their combination simultaneously targeted both renal and muscular outcomes. Furthermore, SGLT2i itself may improve skeletal muscle mitochondrial function and exercise endurance, providing mechanistic support for the ‘pharmacotherapy-exercise' combined strategy. Additionally, this combined therapeutic approach exerts a positive impact on patients' overall health status, as reflected by improved post-exercise heart rate and enhanced exercise tolerance.

### 5.4 Limitations of current evidence

Currently, research specifically investigating SGLT2 inhibitor combined with exercise therapy for DKD remains relatively limited, and the modest number of retrieved studies reflects that direct investigation of combined SGLT2i and exercise intervention in the DKD field is still in its early stages. In some studies, exercise served only as a background intervention or a component of a comprehensive management strategy rather than the primary interventional factor. The majority of basic research has employed animal models, and clinical evidence still requires further validation through additional randomized controlled trials. Different SGLT2i agents (empagliflozin, dapagliflozin, tofogliflozin, ipragliflozin, etc.) exhibit differences in pharmacokinetics and tissue specificity; the effects of combined exercise may vary by drug class, and data remain limited. Nevertheless, limited observational data suggest that this combined therapy may confer complementary renoprotective effects. As illustrated in Figure 2, SGLT2 inhibitors combined with exercise may confer renal benefits through the following mechanisms: (1) optimizing renal hemodynamics and function — lowering blood pressure, improving hemodynamic profiles, and reducing renal workload; (2) improving glycemic and metabolic parameters — reducing blood glucose, body weight, and insulin resistance; (3) attenuating inflammation and oxidative stress — mitigating inflammatory and oxidative responses and modulating immune function; and (4) providing cardiovascular benefits — reducing the risk of cardiovascular events.

**Figure 2:**
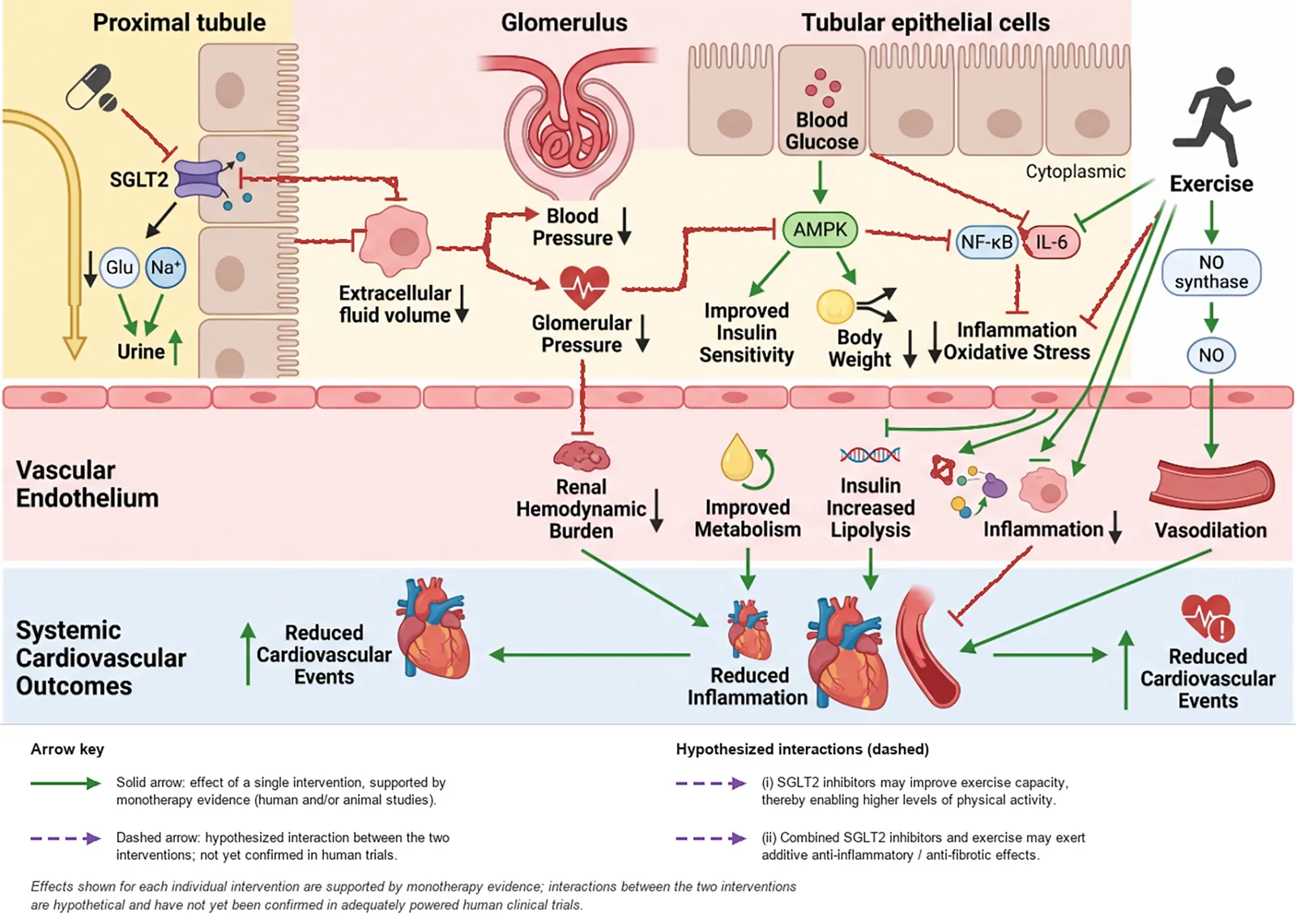
Potential complementary effects of SGLT2 inhibitors and exercise on renal and cardiovascular health. Combined treatment may improve renal hemodynamics and function, enhance glucose metabolism and insulin sensitivity, and reduce body weight. It may also attenuate inflammation and oxidative stress while providing cardiovascular protection. These complementary effects may collectively reduce renal burden and the risk of cardiovascular disease.

## 6 Safety and tolerability assessment

### 6.1 Risk of ketoacidosis and hypoglycemia

In the treatment of DKD, the combined application of SGLT2 inhibitors has demonstrated favorable safety and tolerability profiles; however, attention must still be directed toward hypoglycemia and exercise-related risks. According to relevant studies, SGLT2 inhibitors exhibit good safety in patients with chronic diseases, particularly when analyzed using large-scale randomized trial data. Results showed that compared with placebo, SGLT2 inhibitors significantly reduced the risk of acute kidney injury (relative risk 0.75) (58). Furthermore, although the use of SGLT2 inhibitors in patients with diabetes increased the risk of ketoacidosis (RR 2.57) and genital tract infections (RR 3.75), no significant increase was observed in severe hypoglycemia (RR 0.86) (59, 60). In the context of exercise therapy, the effects of combining SGLT2 inhibitors with exercise also require careful evaluation. The benefits of exercise therapy for patients with diabetes are well established; however, the potential risk of hypoglycemia must be considered when implementing exercise programs. Existing studies indicate that exercise training can improve cardiovascular function and metabolic health in patients with diabetes, and routine moderate-intensity exercise does not require temporary discontinuation of SGLT2 inhibitor therapy. Rare cases of euglycemic diabetic ketoacidosis have been reported in SGLT2 inhibitor-treated patients under conditions of metabolic stress (46). International consensus recommendations on SGLT2 inhibitor use during periods of physiological stress advise withholding these agents during acute illness, significant dehydration, or prolonged fasting, and before major surgery (typically at least 3 days) (61). For exceptionally prolonged or high-intensity endurance activities, or when exercise is undertaken during fasting, carbohydrate restriction, or intercurrent illness, the risk of euglycemic diabetic ketoacidosis and volume depletion increases, and individualized decisions regarding temporary withholding should be made in consultation with the treating clinician. In elderly patients with heart failure, SGLT2 inhibitors have shown acceptable safety and tolerability in real-world practice (62). Nevertheless, exercise-related hypoglycemic events still require close monitoring (55). Murillo et al. (63) demonstrated in an experimental study that high-intensity interval training (HIIT) can improve glycemic stability and reduce hypoglycemic episodes compared with traditional moderate-intensity continuous training (MICT).

### 6.2 Personalized treatment and exercise prescription

Furthermore, the formulation of exercise intervention protocols must consider individual circumstances. Exercise prescriptions should be tailored according to individual characteristics, adhere to the principles of exercise science, and be adjusted promptly based on actual conditions to ensure the safe, effective, and sustainable implementation of patients' fitness activities (53, 64). The evidence-based exercise prescription framework is summarized in Table 3.

**Table 3:** Exercise prescription for patients with diabetic kidney disease (DKD) according to the FITT (frequency, intensity, time, type) principle.

FITT component	Aerobic exercise	Resistance exercise
Frequency	Spread across the week: ≥5 days/week of moderate-intensity activity or ≥3 days/week of vigorous activity (or an equivalent combination), consistent with ADA and physical-activity guidelines for diabetes.	2–3 sessions/week on non-consecutive days.
Intensity	Moderate intensity by default (Borg rating of perceived exertion 11–13, or 40%–59% of heart-rate reserve); vigorous intensity as tolerated. Interpret heart-rate targets cautiously, as chronotropic responses may be blunted by autonomic neuropathy, beta-blockade, or advanced CKD; cardiopulmonary exercise testing (CPET)-guided prescription where available.	Initially moderate loads with progressive overload, reflecting evidence of improved insulin sensitivity and muscle mass in CKD populations; intensity individualized by perceived exertion or heart-rate reserve as for aerobic exercise.
Time	≥150 min/week of moderate-intensity activity (or ≥75 min/week of vigorous activity, or an equivalent combination), accumulated in bouts of ≥10 min.	Typically 1–3 sets of 10–15 repetitions per exercise (per standard resistance-training guidelines).
Type	Rhythmic, large-muscle-group activities; brisk walking and cycling are practical defaults.	8–10 exercises targeting all major muscle groups.
Dose individualization and monitoring: Prescribe and titrate intensity using rating of perceived exertion (e.g., Borg 11–13) or heart-rate reserve; CPET-guided prescription where available.		
CKD stage adjustment: Lower starting volumes and closer supervision for CKD stage G4–G5 and patients on dialysis; time sessions relative to dialysis; avoid exhaustive isometric exercise in uncontrolled hypertension.		
Pre-participation screening and contraindications: Evaluate for unstable cardiovascular disease, uncontrolled hypertension (>180/110 mmHg), acute illness, and recent AKI before initiation; align sick-day rules with SGLT2 inhibitor use.		
Progression and adherence: Gradual progression with ongoing safety monitoring; adopt strategies addressing the well-known barriers to exercise in this population.		

## 7 Future perspectives

In the clinical practice of SGLT2 inhibitors combined with exercise therapy for DKD, precision medicine necessitates the integration of patients' genetic background, CKD stage, metabolic phenotype, and exercise tolerance to formulate individualized combination regimens. Regarding efficacy and safety assessment, the following biomarkers should be systematically monitored: urinary TGF-*β*1 (reflecting the degree of renal fibrosis), NGAL/KIM-1 (evaluating tubular injury), urinary albumin-to-creatinine ratio UACR (assessing glomerular injury), and serum cystatin C (estimating eGFR changes). The optimization of exercise prescription requires comprehensive consideration of CKD stage, cardiovascular status, and drug response, with individualized adjustment of exercise type (aerobic/resistance/HIIT), intensity, frequency, duration, and safety monitoring strategies, in order to maximize exercise benefits while minimizing the risks of dehydration, hypoglycemia, and ketoacidosis. Furthermore, this field currently lacks high-quality prospective randomized controlled trials directly comparing the effects of 'SGLT2i combined with exercise' versus SGLT2i alone or exercise alone on renal hard endpoints in DKD (eGFR decline rate, progression to ESKD, changes in albuminuria), representing the most critical research gap and future direction. There is an urgent need for translational research to convert the molecular mechanisms of potentially additive anti-fibrotic and renoprotective effects of SGLT2i and exercise revealed in basic research (such as discoveries of TGF-*β*1/Smad and AMPK pathways in animal models) into diagnostic tools, therapeutic strategies, and exercise prescriptions applicable to clinical DKD patients, thereby achieving the ‘bench to bedside' transition.

Design considerations for such a definitive trial are as follows. A multicenter, randomized, controlled study should enroll patients with type 2 diabetes and albuminuria-defined DKD (e.g., eGFR 20–75 mL/min/1.73 m^2^ with UACR 200–5000 mg/g) already receiving maximal tolerated RAS blockade, randomized in a 2 × 2 factorial design to SGLT2 inhibitor and supervised exercise, yielding four arms: combined therapy, SGLT2 inhibitor alone, exercise alone, and usual care. The exercise intervention should follow a standardized, prespecified FITT (frequency, intensity, time, type) protocol—for example, moderate-intensity aerobic exercise at 40%–59% heart-rate reserve plus progressive resistance training, 3–5 sessions per week for at least 12 months—with attendance, adherence, and intensity documented to allow dose–response analyses. The primary endpoint should be a validated hard composite such as sustained ≥40% eGFR decline, kidney failure (dialysis or transplantation), or renal or cardiovascular death, with annualized eGFR slope and percentage change in UACR as key secondary endpoints. Given expected annual eGFR declines of 3–5 mL/min/1.73 m^2^ in this population, a trial of 24–36 months' duration with approximately 300–500 participants per arm would be required to detect a clinically meaningful between-group difference. Safety monitoring should include pre-specified definitions of euglycemic diabetic ketoacidosis, volume depletion, and exercise-related adverse events.

## 8 Conclusion

The pathogenesis of diabetic kidney disease is complex and multifactorial, encompassing hemodynamic, metabolic, inflammatory, and fibrotic processes that can be addressed through comprehensive, multi-dimensional management strategies. SGLT2 inhibitors and exercise each act on several of these pathways through partially distinct and complementary mechanisms, providing a biologically plausible basis for their combined use. However, direct clinical evidence demonstrating that combined SGLT2 inhibitor and exercise therapy is superior to either intervention alone remains limited, and no adequately powered randomized controlled trial has yet directly compared combined therapy with monotherapy on hard renal endpoints in DKD. Until such trials are completed, the combined approach should be regarded as a promising but unproven strategy, and clinical management should continue to rely on the established benefits of each intervention individually. In summary, the comprehensive management of DKD requires a balance between pharmacological and non-pharmacological interventions. We must emphasize the multiple mechanisms of action of SGLT2 inhibitors while not overlooking the potential of exercise therapy in improving patients' quality of life and metabolic status. By strengthening future clinical research and multidisciplinary collaboration, and promoting the development of precision individualized treatment strategies, we anticipate achieving major breakthroughs in DKD management, ultimately improving patients' quality of life and alleviating the socioeconomic burden imposed by this disease.

## Author contributions

HL: Conceptualization, Data curation, Formal analysis, Investigation, Methodology, Writing – original draft, Writing – review & editing. MA: Conceptualization, Data curation, Formal analysis, Writing – original draft. XH: Conceptualization, Data curation, Writing – original draft. CL: Conceptualization, Data curation, Writing – review & editing. XJ: Funding acquisition, Resources, Supervision, Validation, Visualization, Writing – original draft, Writing – review & editing.
